# Incidence and impact of food aversions among patients with cancer receiving outpatient chemotherapy: a one-year prospective survey

**DOI:** 10.1007/s00520-024-09028-7

**Published:** 2024-11-20

**Authors:** Machi Suka, Atsushi Katsube, Reiko Fujimoto, Tadashi Uwagawa, Takashi Shimada, Shingo Yano, Takashi Yamauchi, Hiroyuki Yanagisawa

**Affiliations:** 1 Department of Public Health and Environmental Medicine, The Jikei University School of Medicine, 3-25-8 Nishi-Shimbashi, Minato-Ku, Tokyo 105-8461 Japan; 2 Division of Clinical Oncology and Hematology, Department of Internal Medicine, The Jikei University School of Medicine, Tokyo, Japan; 3 The Jikei University Hospital, Tokyo, Japan; 4 The Jikei University School of Medicine, Tokyo, Japan

**Keywords:** Chemotherapy, Food aversions, Malnutrition, Incidence, Outpatients, Prospective study

## Abstract

**Purpose:**

To determine the current incidence and impact of chemotherapy-associated food aversions in a variety of cancer types.

**Methods:**

Cancer patients aged 18 years and older who received chemotherapy infusions at the outpatient chemotherapy unit of a university hospital between May 2022 and April 2023 were included in the study (*n* = 243). To monitor the occurrence of food aversions, participants were asked to complete a food preference questionnaire each time they visited the outpatient chemotherapy unit.

**Results:**

During the one-year survey period, one in four cancer patients receiving outpatient chemotherapy developed food aversions, and one in four of them complained of interference with daily life due to eating problems at the same time or later. The median time to the onset of food aversion was 46 (interquartile range 36–77) days after the start of chemotherapy. The incidence of food aversions was significantly higher in patients who were women, had a digestive, gynecologic, or breast cancer, and received more cytotoxic agents in chemotherapy. Patients who developed food aversions tended to lose more body weight than those who did not.

**Conclusion:**

Food aversions were still common among cancer patients undergoing chemotherapy. Even an aversion to a single food may have affected the patient’s nutritional status. Healthcare professionals should closely monitor the occurrence of food aversions, especially in the early days of chemotherapy induction, to detect an increasing risk of malnutrition.

**Trial registration:**

Not applicable.

**Supplementary Information:**

The online version contains supplementary material available at 10.1007/s00520-024-09028-7.

## Background

In recent years, the treatment setting for cancer patients has shifted from inpatient to outpatient care. Many cancer patients receive chemotherapy primarily on an outpatient basis. Patients undergoing chemotherapy often experience nutrition-related side effects such as loss of appetite, taste changes, and food aversions [1]. The occurrence of nutrition-related side effects can lead to inadequate nutrition intake and unintentional weight loss [2, 3]. Patients experiencing nutrition-related side effects need advice and support to prevent or eliminate nutritional deficiencies, achieve or maintain a healthy weight, maximize quality of life, and improve prognosis [4].

Food aversion is the phenomenon of avoiding a particular food that one used to eat without any concern, triggered by an unpleasant experience, and is distinct from taste and smell disorders [5–7]. Food aversions associated with chemotherapy have been studied since 1980s [8]. Previous studies have shown that food aversions mainly occurred around the first and second chemotherapy infusions [9] or an average of 30–60 days after chemotherapy induction [10]. Those who experienced digestive side effects such as nausea and vomiting were more likely to report food aversions [8]. On the other hand, several researchers have reported that when patients were offered a novel food as a scapegoat within an hour before the start of chemotherapy, they developed an aversion only to the novel food and not to their usual diet [11–13]. It is generally believed that the experience of unpleasant side effects in the early days of chemotherapy induction may serve as a conditioned stimulus that causes food aversions [7, 8]. However, not all patients who experienced unpleasant side effects from chemotherapy developed food aversions [9, 14, 15]. Food aversions became less noticeable in some patients a few months after onset, but persisted in others [9, 10]. The detailed mechanisms of chemotherapy-associated food aversions are not yet fully understood, nor are the predictors of which patients are more likely to develop food aversions clearly elucidated.

Recent advances in anticancer therapy and supportive care may have altered the incidence and impact of food aversions in cancer patients [16, 17]. Unfortunately, to the best of our knowledge, there have been few recent research reports on this topic. A better understanding of food aversions based on more recent data is needed to provide appropriate advice and support to cancer patients. We conducted a one-year prospective survey to examine the incidence of food aversions among cancer patients undergoing outpatient chemotherapy at a university hospital. To assess the impact of the occurrence of food aversions on nutritional status, changes in body weight were monitored after the start of chemotherapy. This study is the first to report on the current incidence and impact of chemotherapy-associated food aversions in a variety of cancer types. The results will show that food aversions were still common among cancer patients undergoing chemotherapy; the incidence of food aversions was significantly higher in patients who were women, had a digestive, gynecologic, or breast cancer, and received more cytotoxic agents in chemotherapy; and patients who developed food aversions tended to lose more body weight than those who did not.

## Methods

### Participants

The Jikei University Hospital is located in the central area of Tokyo, Japan and provides comprehensive medical services with 1,075 beds and 2,600 outpatients per day. The hospital has been designated by the Japanese Ministry of Health, Labour, and Welfare as a Regional Base Hospital for Cancer Treatment. A cumulative total of 10,000 cancer patients annually visit the outpatient chemotherapy unit to receive chemotherapy infusions. We conducted a one-year prospective survey of service users of the outpatient chemotherapy unit to determine the current incidence and impact of chemotherapy-associated food aversions in a variety of cancer types. The study protocol was approved by the Ethics Committee of the Jikei University School of Medicine (reference number 33–423(11,048)) and has been conducted in accordance with the Ethical Guidelines for Medical and Health Research Involving Human Subjects by the Japanese Government.

Inclusion criteria for the study were adults 18 years of age or older who were diagnosed with cancer, had an Eastern Cooperative Oncology Group (ECOG) performance status of grade 2 or higher [18], and received chemotherapy infusions at the outpatient chemotherapy unit between May 2022 and April 2023. All eligible patients received information about the study protocol when attending the pre-treatment chemotherapy orientation. Only those who voluntarily agreed to participate in the survey signed an informed consent form. Participants were asked by the nurse to fill out a food preference questionnaire (one sheet of paper) each time they visited the outpatient chemotherapy unit. They completed the questionnaire during their chemotherapy infusion, which took only a few minutes.

During the one-year survey period, 1,086 cancer patients received chemotherapy infusions at the outpatient chemotherapy unit. Of these, 581 (53.5%) agreed to participate in the survey. Previous studies have shown that most food aversions occur early in chemotherapy induction [9, 10]. Therefore, of the 581 respondents, 243 patients whose first response to the questionnaire was within 12 weeks of the date of first chemotherapy infusion were included in the study. The observation period totaled 22,349 patient-days. The median number of visits to the outpatient chemotherapy unit and median number of responses to the questionnaire for each patient were 7 (interquartile range 5–11) and 4 (interquartile range 3–6), respectively.

As will be shown later, this study found that one in four cancer patients receiving outpatient chemotherapy developed food aversions. Assuming a true difference of 1 kg in mean weight change between patients with and without food aversions, the sample size required to reject the null hypothesis of equal mean weight change in the two groups was estimated to be 168 (42 cases and 126 controls) with an alpha error of 0.05 and a beta error of 0.20. The actual number of study participants was larger than the estimated sample size, suggesting that the difference in mean weight change between patients with and without food aversions would be detectable.

### Measures

Our preliminary literature review did not reveal any established screening methods for the onset of food aversion. We needed to develop an original questionnaire that would detect the occurrence of food aversions efficiently while minimizing the burden on the patient in answering the questions. The food preference questionnaire used in the survey asked about the patient's 1) interference with daily life due to eating problems, 2) food aversions, and 3) favorite foods in the last week. The first question asked whether the patient had any eating problems that interfered with daily life. The second question asked whether the patient came to dislike and avoid eating a particular food after the start of chemotherapy, and if yes, further asked what and why the patient avoided eating it (stomatitis / dry mouth / taste / smell / unidentified causes / others). Participants were instructed not to answer ‘yes’ to this question if they were refraining from eating it on doctor's orders. The third question asked whether there were any foods that became favorites after the start of chemotherapy. Those who answered ‘yes’ to the second question and chose ‘taste’, ‘smell’, ‘unidentified causes’ or ‘others’ as the reason for avoidance were counted as having developed a food aversion. To distinguish aversions to particular foods from taste and smell disorders, the responses to the second question were carefully examined one by one. Those who reported avoiding ‘almost all types of food’ were excluded from the cases because they were more likely to be affected by events other than food aversion.

Demographic and clinical characteristics of the study participants, as well as their body weight at each visit, were collected from their electronic medical records. Cancer types registered according to the International Statistical Classification of Diseases and Related Health Problems 10th Revision (ICD-10) were classified into four groups: lip, oral cavity and pharynx (C00-C14), digestive organs (C15-C26), larynx (C32), and peritoneum (C48) were “Digestive”; breast (C50) and female genital organs (C51-C58) were “Gynecologic/Breast”; respiratory and intrathoracic organs (C33-C39) and lymphoid, hematopoietic and related tissue (C81-96) were “Lung/Blood”; and nasal cavity (C30), accessory sinuses (C31), skin (C43-44), connective and soft tissue (C49), male genital organs (C60-63), urinary tract (C64-68), eye, brain and other parts of central nervous system (C69-C72), and thyroid and other endocrine glands (C73-75) were “Others”. Chemotherapy used cytotoxic agents (antimetabolites, alkylating agents, topoisomerase inhibitors, microtubule inhibitors, platinum-based agents, antibiotics, and prednisolone), molecularly targeted agents (kinase inhibitors, monoclonal antibodies, and immune checkpoint inhibitors), or both at the discretion of the expert teams. Body weight was measured by the patients themselves when they came to the outpatient chemotherapy unit using an automatic scale, and the value was verified by the nurse. Weight changes from the date of first chemotherapy infusion were calculated at each visit.

### Incidence rates of eating complaints

The incidence rates of food aversions and interference with daily life due to eating problems were calculated as the number of patients who reported the complaints, respectively during the survey period, divided by the total amount of time at risk (i.e. the number of days when the outcome could have occurred, patient-days). The time at risk for each patient was from the date of first chemotherapy infusion to the date of the first reported complaint or, in cases with no complaints, to the date of the last response to the food preference questionnaire.

### Statistical analysis

All statistical analyses were performed using the SAS ver. 9.4 (SAS Institute, Cary, NC, USA). Significant levels were set at *p* < 0.05. Kaplan-Mayer survival analysis with Log-rank test was performed to compare the cumulative incidence of food aversions. Cox proportional hazard model was used to calculate the hazard ratios (HRs) with 95% confidence intervals (CIs) for developing food aversions with adjustment for gender, age (in Model 1), and cancer type (in Models 2 and 3). The percentage of patients with eating problems that interfered with their daily life was compared using Fisher’s exact test. Multiple logistic regression was performed to examine the association between food aversions and interference with daily life due to eating problems and calculate the odds ratios (ORs) adjusted for gender and age. The mean weight changes from the date of first chemotherapy infusion were compared using repeated measure analysis of variance.

## Results

Table 1 shows the characteristics of study participants. About half of the participants were female and over 65 years old. The majority had recommended weight (body mass index 18.5–24.9) at the start of chemotherapy. All common cancer types were covered, including 33 breast cancers, 32 lymphoma, 22 pancreatic cancers, 22 ovarian cancers, 19 lung cancers, 16 gastric cancers, 13 rectal cancers, and 10 colon cancers. The most frequently used chemotherapy agents were microtubule inhibitors (*n* = 91), followed by alkylating agents (*n* = 79), platinum-based agents (*n* = 79), antimetabolites (*n* = 78), monoclonal antibodies (*n* = 71), topoisomerase inhibitors (*n* = 59), immune checkpoint inhibitors (*n* = 53), prednisolone (*n* = 23), kinase inhibitors (*n* = 5), and antibiotics (*n* = 2). No patients received concomitant radiation therapy.

**Table 1:** Characteristics of study participants

		All	Cancer type			
			Digestive (C00-C26, C32, C48)	Gynecologic/Breast (C50-C58)	Lung/Blood (C33-C31, C81-C96)	Others
N		243	86	75	51	31
Gender	Men	121	60	1	36	24
		49.8%	69.8%	1.3%	70.6%	77.4%
	Women	122	26	74	15	7
		50.2%	30.2%	98.7%	29.4%	22.6%
Age	18–64 years	126	30	65	19	12
		51.9%	34.9%	86.7%	37.3%	38.7%
	65 + years	117	56	10	32	19
		48.1%	65.1%	13.3%	62.7%	61.3%
Body Mass Index	Underweight (< 18.5)	39	12	17	8	2
		16.0%	14.0%	22.7%	15.7%	6.5%
	Normal (18.5–24.9)	148	58	40	33	17
		60.9%	67.4%	53.3%	64.7%	54.8%
	Overweight (25.0 +)	56	16	18	10	12
		23.0%	18.6%	24.0%	19.6%	38.7%
Chemotherapy	Cytotoxic agents	129	42	70	5	12
		53.1%	48.8%	93.3%	9.8%	38.7%
	Molecularly targeted agents	48	11	0	21	16
		19.8%	12.8%	0.0%	41.2%	51.6%
	Both	66	33	5	25	3
		27.2%	38.4%	6.7%	49.0%	9.7%

Cancer types were classified by the International Statistical Classification of Diseases and Related Health Problems 10th Revision (ICD-10)

During the observation period, 56 patients (23.0%) reported developing food aversions. The mean number of foods avoided was 1.6 (standard deviation 1.4), ranging from 1 to 7 items. There were 23 patients (41.1%) who avoided two or more foods. The most frequently reported one was rice/bread (*n* = 16), followed by meat (*n* = 15), greasy foods (*n* = 15), fish (*n* = 11), coffee/tea (*n* = 10), and sweets (*n* = 8).

**Fig. 1:**
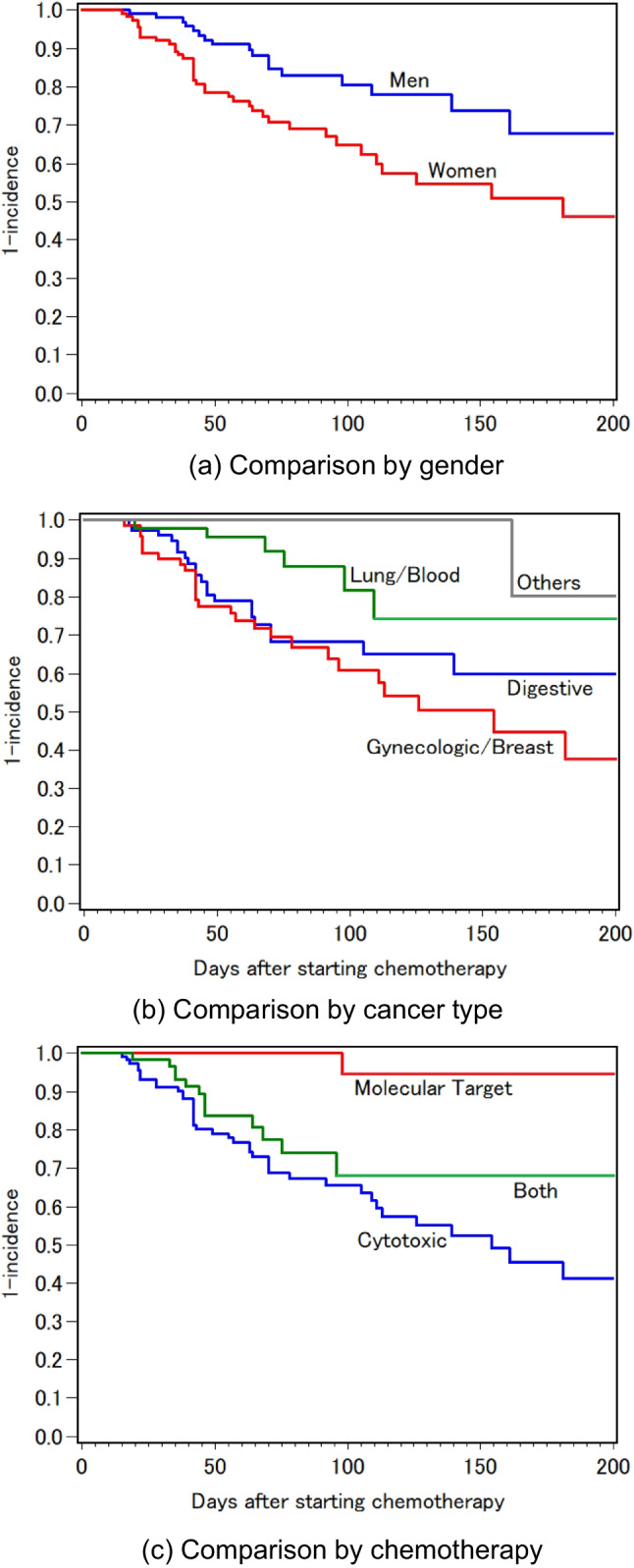
Cumulative incidence of food aversions (Kaplan-Meyer curves)

The median time to the onset of food aversion was 46 (interquartile range 36–77) days after the start of chemotherapy. Individual time courses for incidence of eating problems were shown in Supplementary file 1. Once patients started to avoid particular foods, they continued to do so throughout the rest of the survey. The incidence rate of food aversions was estimated at 2.9 per 1000 patient-days. This rate was higher in women (*p* = 0.006, Fig. 1a), lower in those with Lung/Blood or Other cancer (*p* < 0.001, Fig. 1b), and lower in those who received only molecularly targeted agents (*p* < 0.001, Fig. 1c). There were no significant differences by age (*p* = 0.362) or body mass index (*p* = 0.555).

Table 2 shows the results of the Cox proportional hazard models to examine the factors associated with food aversions. After adjustment for gender and age, the HR for Lung/Blood cancers was significantly lower than that for Digestive cancers (Model 1). Further adjusting for cancer type, the HR for molecularly targeted agents was significantly lower than that for cytotoxic agents (Model 2), and the magnitude of risk increased in proportion to the number of cytotoxic drugs (Model 3). When analyzed by type of cytotoxic agents, significantly higher HRs were observed in antimetabolites (HR 6.44, 95%CI 1.38–52.99) and microtubule inhibitors (HR 2.36, 95%CI 1.00–6.15).

**Table 2:** Factors associated with food aversions

		Model 1		Model 2		Model 3	
		HR	95%CI	HR	95%CI	HR	95%CI
Gender	Men	1.00	ref	1.00	ref	1.00	ref
	Women	1.89	0.89–3.92	1.86	0.85–4.00	1.61	0.76–3.36
Age	18–64 years	1.00	ref	1.00	ref	1.00	ref
	65 + years	1.17	0.61–2.26	1.27	0.67–2.41	1.29	0.67–2.49
Cancer type	Digestive (C00-C26, C32, C48)	1.00	ref	1.00	ref	1.00	ref
	Gynecologic/Breast (C50-C58)	0.94	0.43–2.20	0.74	0.32–1.82	1.05	0.47–2.44
	Lung/Blood (C33-C31, C81-C96)	0.38	0.14–0.88	0.59	0.21–1.45	0.24	0.08–0.63
	Others	0.12	0.01–0.56	0.23	0.01–1.16	0.29	0.02–1.49
Chemotherapy	Cytotoxic agents	ー		1.64	0.81–3.52	ー	
	Molecularly targeted agents	ー		0.14	0.01–0.73	ー	
	Both	ー		1.00	ref	ー	
	Number of Cytotoxic agents	ー		ー		2.51	1.50–4.66

The hazard ratios (HR) and 95% confidence intervals (CI) for developing food aversions were calculated using Cox proportional hazard models with adjustment for gender, age, (in Model 1), and cancer type (in Models 2 and 3)

During the observation period, 33 patients (13.6%) reported developing eating problems that interfered with their daily life. The incidence rate of interference with daily life due to eating problems was estimated at 1.6 per 1000 patient-days. Of the 56 patients with food aversions, 25.0% complained of interference with daily life due to eating problems at the same time or later. This percentage was significantly greater than that in the 187 patients without food aversions (10.2%, *p* = 0.005). In multiple logistic regression adjusted for gender and age, the OR of food aversions for developing interference with daily life due to eating problems was estimated to be 2.81 (95%CI 1.27–6.22). The magnitude of risk was not related to the number of foods avoided.

Table 3 shows the mean weight changes from the date of first chemotherapy infusion in 207 patients for whom follow-up weight data were available. In the analysis over the survey period, the patients with food aversions showed a tendency to lose weight, but the patients without food aversions did not. When analyzed by time period, this difference was consistently observed, although not statistically significant. The degree of weight loss associated with food aversions was not related to the number of foods avoided, nor was it related to which foods were being avoided.

**Table 3:** Mean weight changes from the date of first chemotherapy infusion

Weeks after starting chemotherapy		Patients without food aversions	Patients with food aversions	p
All time period	Number of responses	707	243	
	Mean (SD)	+ 0.08 (2.66)	−0.87 (2.93)	0.015
1–8 weeks	Number of responses	238	69	
	Mean (SD)	+ 0.03 (1.38)	−0.19 (1.22)	0.385
9–16 weeks	Number of responses	203	61	
	Mean (SD)	+ 0.06 (2.57)	−0.72 (1.95)	0.098
16–24 weeks	Number of responses	138	50	
	Mean (SD)	+ 0.14 (3.38)	−0.71 (3.19)	0.077
24 + weeks	Number of responses	128	63	
	Mean (SD)	+ 0.14 (3.58)	−1.90 (4.31)	0.220

The simple average (Mean) and standard deviation (SD) of weight changes (kg) from the date of first chemotherapy infusion were calculated in 207 patients for whom follow-up weight data were available. P values were determined by repeated measures analysis of variance

## Discussion

A one-year prospective survey was conducted to examine the incidence of food aversions among cancer patients undergoing outpatient chemotherapy at a university hospital. Since all service users of the outpatient chemotherapy unit were invited to participate in the survey, the study participants covered all common cancer types. The responses to the food preference questionnaire were carefully reviewed to accurately identify cases of food aversions. These allowed us to find significant differences in food aversion formation by gender, cancer type, and chemotherapy agent. We also found that patients who developed food aversions tended to lose more body weight than those who did not. Most previous studies have not considered the influence of cancer site or type of chemotherapy agent on food aversion formation [8]. There have been few research reports on the impact of food aversions on nutritional status or body weight. The results of this study provide valuable data of the current incidence and impact of chemotherapy-associated food aversions in a variety of cancer types.

During the one-year survey period, one in four cancer patients receiving outpatient chemotherapy developed food aversions, and one in four of them complained of interference with daily life due to eating problems at the same time or later. The prevalence of food aversion in patients undergoing cancer treatment has been reported to range from 21 to 62% [8]. The percentage of patients with food aversions in this study (23.0%) was near the lower end of the range of values reported in previous studies. Recently, conventional cytotoxic agents in chemotherapy have been increasingly replaced by novel molecularly targeted agents. With the changes in chemotherapy agents, the incidence of adverse events has been on the decline [19]. On the other hand, the management of side effects of chemotherapy has improved considerably to date [16, 17]. The advances in anticancer therapy and supportive care may also have contributed to the decline in the incidence of food aversions.

Although the incidence of chemotherapy-associated food aversions may have decreased, it remains high. Once patients started to avoid particular foods, their aversions were likely to persist throughout the course of chemotherapy, and their body weight was likely to gradually decrease. Early detection of food aversion can provide an opportunity to prevent malnutrition and interference with daily life. The median time to the onset of food aversion was 46 days after the start of chemotherapy, which is consistent with previous studies showing that food aversions mainly occurred around the first and second chemotherapy infusions [9] or an average of 30–60 days after chemotherapy induction [10]. It may be recommended that healthcare professionals closely monitor the occurrence of food aversions, especially in the early days of chemotherapy induction.

The incidence of food aversions was significantly higher in patients with Digestive or Gynecologic/Breast cancer and in those who received more cytotoxic agents in chemotherapy. A few studies have compared the incidence of food aversions between different cancer types or chemotherapy agents. Mattes et.al. found no obvious differences in the incidence of food aversions between breast and lung cancer patients [14], but their study was conducted more than 30 years ago and had a small sample size. Coa et.al. reported that food aversions tended to be more prevalent in patients with gastrointestinal and breast cancers than in patients with lung, other solids, and hematologic cancers [3], which is consistent with the results of this study. There are several possible reasons why Gynecologic/Breast cancer patients had a high risk comparable to that of Digestive cancer patients. One reason is that all the patients with Gynecologic/Breast cancer received cytotoxic agents. Digestive, Lung/Blood, and Other cancers were more likely to be treated with molecularly targeted agents, which cause fewer serious adverse events. Another reason is that almost all the patients with Gynecologic/Breast cancer were women. Previous human and animal studies have revealed that sex differences in taste-guided behavior and food choice may be attributed to sex hormones [7, 20, 21]. On the other hand, since women are the main meal preparers in Japan, they are more likely to perceive changes in taste, aroma, and texture during meal preparation, leading to changes in food preferences. It seems that both biological and sociological mechanisms are involved in gender differences. Healthcare professionals should be aware of the high risk of food aversion in gynecologic and breast cancer patients treated with multiple cytotoxic agents.

In this study, our original questionnaire was used to identify cases of food aversions. It took only a few minutes for the patient to answer. To date, there are no established screening methods for the onset of food aversion. The food preference questionnaire has the potential to be a useful tool for detecting the occurrence of food aversions without causing any harm to the patient. Further studies are needed to verify the reliability and validity of the questionnaire before it can be recommended as a standard screening tool for food aversions.

This study is unique in determining the incidence and impact of chemotherapy-induced food aversions in different types of cancer based on the most recent data. The results of this study are useful for understanding the nutritional issues often faced by cancer patients undergoing chemotherapy. On the contrary, this study has the following potential limitations. First, follow-up data were only available on the day of the patient’s visit to the outpatient chemotherapy unit. Participants were free to decide whether or not to answer the questionnaire each time. If a participant was not feeling well or unwilling to do so, he or she did not respond that time. The exact date of onset of food aversion may not have been accurately recorded. The number of days from the start of chemotherapy to the occurrence of food aversions may actually have been shorter than reported in this study. Second, information on the occurrence of food aversions was collected using a self-administered questionnaire. We cannot completely rule out the possibility that the occurrence of food aversions was underreported or overlooked in some patients. The incidence rate of food aversions reported in this study may have been underestimated. Third, participants were followed only during outpatient chemotherapy. Once patients started to avoid particular foods, they continued to do so throughout the rest of the survey. However, it was unknown whether the food aversions would continue after chemotherapy ended. Fourth, this study was conducted at one university hospital. It is unlikely that the study participants represent all cancer patients undergoing outpatient chemotherapy in Japan. Furthermore, it is not certain whether the findings from this study are applicable to countries other than Japan. Further studies are needed to confirm the generalizability of the findings.

## Conclusion

The one-year prospective survey of service users of the outpatient chemotherapy unit demonstrated the current incidence and impact of chemotherapy-associated food aversions in a variety of cancer types. Although the incidence of food aversions may have decreased with recent changes in chemotherapy agents, one in four cancer patients undergoing outpatient chemotherapy developed food aversions. Gynecologic and breast cancer patients, who were more likely to receive multiple cytotoxic agents, had a high risk of food aversion comparable to that of digestive cancer patients. Patients who developed food aversions tended to lose more body weight than those who did not. Healthcare professionals should closely monitor the occurrence of food aversions, especially in the early days of chemotherapy induction, to detect an increasing risk of malnutrition.

## Supplementary Information

Below is the link to the electronic supplementary material.

Supplementary file1 (PDF 74 KB)

## Data Availability

No datasets were generated or analysed during the current study.
