# Supplementary figures and images for: Incidence and impact of food aversions among patients with cancer receiving outpatient chemotherapy: a one-year prospective survey
Source: Support Care Cancer. 2024 Nov 20;32(12):810. doi: 10.1007/s00520-024-09028-7 (PMC11579046; doi:10.1007/s00520-024-09028-7)

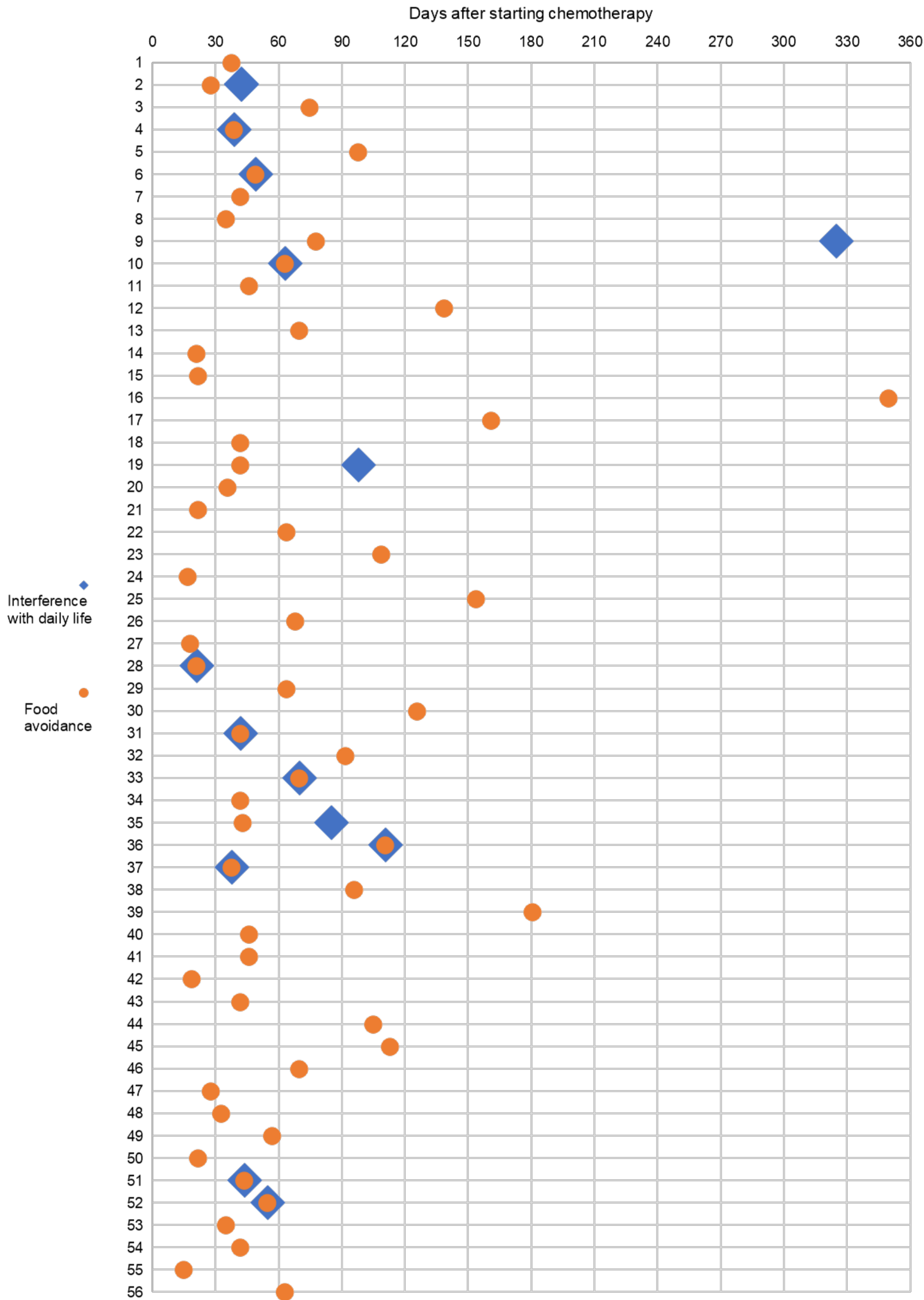

Appendix. Individual time courses for incidence of eating problems

Supplement: Supplementary file 1 — Supplementary file1 (PDF 74 KB) [file 520_2024_9028_MOESM1_ESM.pdf]
